# Waiving Subsequent Complete Lymph Node Dissection in Melanoma Patients with Positive Sentinel Lymph Node Does Not Result in Worse Outcome on 20-Year Analysis

**DOI:** 10.3390/cancers13215425

**Published:** 2021-10-29

**Authors:** Laura Susok, Celine Nick, Jürgen C. Becker, Falk G. Bechara, Markus Stücker, Waldemar Uhl, Thilo Gambichler

**Affiliations:** 1 Department of Dermatology, Venereology and Allergology, Skin Cancer Center, Ruhr-University Bochum, 44801 Bochum, Germany; laura.susok@klinikum-bochum.de (L.S.); celine.nick@klinikum-bochum.de (C.N.); f.bechara@klinikum-bochum.de (F.G.B.); m.stuecker@klinikum-bochum.de (M.S.); 2 Department of Dermatology, Translational Skin Cancer Research, German Cancer Consortium (DKTK) Partner Site Essen/Düsseldorf, University Duisburg-Essen, 47057 Essen, Germany; j.becker@dkfz-heidelberg.de; 3 Translational Skin Cancer Research, DKTK Site Essen, ED03, Deutsches Krebsforschungszentrum (DKFZ), 69120 Heidelberg, Germany; 4 Department of General and Visceral Surgery, Ruhr-University Bochum, 44791 Bochum, Germany; waldemar.uhl@klinikum-bochum.de

**Keywords:** malignant melanoma, sentinel lymph node biopsy, complete lymph node dissection, lymphadenectomy, melanoma-specific survival, micrometastasis

## Abstract

**Simple Summary:**

The aim of the present study was to investigate long-term outcomes of melanoma patients who had micrometastasis on sentinel lymph node (SLN) biopsy. We focused on the comparison between melanoma patients with and without complete lymph node dissection (CLND) following a positive SLN biopsy result. Patients without CLND did not significantly differ from patients with CLND in regard to age, gender, tumor thickness, tumor ulceration, capsule infiltration of SLN, and invasion level of SLN. On 10-year analysis, we did not observe a significantly increased risk for melanoma relapse or melanoma-specific death in patients who did not undergo CLND after the detection of micrometastases on SLN biopsy. On 20-year analysis, again, the patients without CLND had no significantly increased risk of melanoma relapse and worse melanoma-specific survival. Hence, our 10-year survival data confirm the current notion that waiving CLND in SLN-positive patients does not result in clinical disadvantages with respect to melanoma-specific survival. For the first time, we demonstrate on 20-year survival analysis that relapse rates and melanoma-specific survival does not significantly differ between patients with or without CLND on long-term follow-up.

**Abstract:**

Complete lymph node dissection (CLND) following positive sentinel lymph node (SLN) biopsy has been the standard of care for decades. We aimed to study melanoma patients with an emphasis on the outcome of patients with versus without CLND following positive SLN biopsy. SLN-positive patients with or without CLND were compared regarding important prognostic clinical and histological characteristics. Ten-year and 20-year survival curves for melanoma relapse and melanoma-specific survival (MSS) were determined by the Kaplan-Meier method and Cox proportional-hazards regression. We studied 258 patients who had micrometastases in their SLN biopsy. CLND was performed in 209 of 258 patients (81%). Hence, in 49 of 258 patients (19%) with SLN micrometastases, CLND was not performed. These patients did not significantly (*p* > 0.05) differ from patients with CLND in regard to age, gender, tumor thickness, tumor ulceration, capsule infiltration of SLN, or invasion level of SLN. On 10-year analysis, we did not observe a significantly increased risk for melanoma relapse and worse in MSS in patients who did not undergo CLND (hazard ratio: 1.1 (95% CI 0.67 to 1.7) and 1.1 (95% CI 0.67 to 1.9), respectively). On 20-year survival analysis, we confirmed that the risk of melanoma relapse and impaired MSS does not significantly increase in patients without CLND (hazard ratio: 1.2 (95% CI 0.8 to 1.9) and 1.3 (95% CI 0.8 to 2.3), respectively). On 10-year as well as 20-year multivariable follow-up analysis (including several important prognostic factors), Cox proportional-hazards regression showed that the status of CLND did not remain in the regression model (*p* > 0.1). Our 10-year data give conclusive support to previous investigations indicating that waiving CLND in patients with SLN micrometastases does not affect MSS. More importantly, our long-term follow-up data confirm for the first time the 10-year survival data of previous investigations.

## 1. Introduction

Cutaneous melanoma is associated with more than 55,000 deaths per annum worldwide. Despite the advent of novel effective therapies, such as immune and targeted therapy, melanoma remains a life-threatening disease once it cannot be cured by surgery alone [1,2]. Sentinel lymph node (SLN)-biopsy (SLNB) is a proven and reliable prognostic tool. As reported in the second Multicenter Selective Lymphadenectomy Trial (MSLT-2) [3,4], the 10-year melanoma-specific survival (MSS) of patients with intermediate-thickness melanomas (1.2–3.5 mm) was 85.1% for SLN-negative and 62.1% for SLN-positive patients (*p* = 0.0001). Currently, SLNB is performed starting at a Breslow tumor thickness of 1 mm. Moreover, thinner (0.75 mm) melanomas with ulceration, patient-age under 40 years, and increased mitosis index are also recommended for SLNB on an individual basis. In patients with thicker melanomas, 10-year MSS was 64.4% for negative SLN and 48.0% (*p* = 0.03) for positive SLN. Indeed, SLN-status frequently proved to be a strong predictor of MSS [1,2,3,4,5].

In many cancer centers all over the world, the clinical value of CLND for melanoma patients with SLN micrometastases has been put into perspective within the last decade. By contrast, in the current German S3-guideline for the management of melanoma, there is still no definitive statement against CLND in case of micrometastases [1]. Notwithstanding, clinical practice has changed due to recent data obtained from two randomized trials. Faries et al. [5] have shown that, on the one hand, immediate CLND increased the rate of regional disease control and provided prognostic information, but on the other hand, CLND did not increase the MSS rate when compared to the observation group [5]. Leiter et al. [6] also found no difference in survival rates of patients treated with CLND compared with observation only. Consequently, Leiter et al. [6] concluded that CLND should not be recommended in melanoma patients with SLN micrometastases of at least a diameter of 1 mm or smaller [6]. The updated results of the aforementioned trial (7-year survival data) showed similar hazard ratios compared with those at the 3-year analysis [7]. Hence, these results confirmed that immediate CLND in SLN-positive patients is not superior to observation with respect to distant metastasis-free survival, relapse-free survival, and overall survival [7]. Indeed, Bilimoria et al. [8] showed that additional micrometastases are detected only in about 20% of melanoma patients undergoing CLND, indicating that 80% of patients may have unnecessarily been put at risk of surgery complications. Furthermore, CLND is associated with considerably greater morbidity than SLNB alone (23% vs. 5%) [8]. Previous retrospective studies did also reveal that CLND for SLN-positive melanoma patients is not superior to observation [9,10]. In the present article, we report our single center long-term experience in patients who had undergone SLNB, focusing on the question of whether waiving CLND following a positive SLNB is a disadvantage with respect to melanoma relapse and MSS.

## 2. Materials and Methods

### 2.1. Patients

The present investigation was carried out at the Skin Cancer Center of the Ruhr-University Bochum (North-Rhine-Westphalia, Germany). The study was approved by the local ethics review board (Ruhr-University Bochum). We selected all melanoma patients who had undergone SLNB between 1999 and 2020 and checked their files for sufficient data with respect to time of primary surgery, SLNB and, if performed, CLND as well as tumor characteristics, such as Breslow thickness, ulceration, SLN status, CLND status, and further clinical follow-up information. Patients with available clinical history and data were included in further analyses. All melanomas were diagnosed by full primary excision with further histological examination. Predominant indication for SLNB was a Breslow tumor thickness of 1 mm or more. For tumors with a thickness between 0.75 mm and 1 mm, a SLNB was considered at presence of ulceration, increased mitotic rate, and age under 40 years. SLNB and CLND were performed in accordance with previous guidelines [1]. As in our previous study [11,12], macro-metastases in regional lymph nodes and distant metastatic disease were checked by physical examination and staging procedures, including ultrasound, computed tomography, and magnetic resonance imaging [1]. Based on previous clinical practice, patients with micrometastases in the SLN usually received CLND. Patients without micrometastases in their SLN and a primary tumor thickness of ≥1.5 mm were usually treated with adjuvant low-dose interferon alfa-2a therapy (Roferon; Roche Pharma AG, Grenzach-Wyhlen, Germany) [1]. Adjuvant high-dose interferon alfa-2b (Intron; MSD, Munich, Germany) was recommended for patients with micro-metastases in their SLN. Follow-up was performed according to the respective national guidelines: For patients with primary tumors <1 mm tumor thickness, clinical investigations were carried out every six months; patients with thicker primary melanoma had their check-ups every three months. Lymph node ultrasound and determination of serum S100B and lactate dehydrogenase were performed as well. In stage III with regional metastatic disease, whole-body imaging was usually performed in 6-month intervals; in stage IV patients, surveillance was scheduled individually [1]. Patient data was retrieved from the electronic database of the hospital; survival data were updated using chart reviews and by contacting patients, relatives, online obituary notices, resident practitioners, oncologists, and dermatologists.

### 2.2. Histology and Immunohistochemistry

Preparation, macroscopic examination, sampling, and microscopic examination were performed in line with the recommendations for pathologic examination of the SLN of melanoma patients as proposed by Scolyer et al. [13] All SLNs were serially sectioned and stained with hematoxylin and eosin. In addition, immunohistochemical staining was performed for S100 and Melan-A/MART-1 (Dako, Hamburg, Germany). All SLNs and nodes from CLND were assessed by two senior dermatopathologists.

### 2.3. Statistics

Data analysis was performed using the statistical package MedCalc^®^ v20.008 (MedCalc Software Ltd., Ostende, Belgium). Distribution of data was assessed by the D’Agostino-Pearson test. Non-normally distributed data were expressed as medians and range. Data were analyzed using the Chi^2^ test and Mann-Whitney test. On univariable analysis, 10-year as well as 20-year survival regarding melanoma relapse and MSS were examined by using the Kaplan–Meier method; differences between the curves were assessed by the log-rank test including hazard ratios and its 95% confidence interval (CI). Multivariable analysis, including logistic regression and Cox proportional-hazards regression, was performed including important prognostic factors (tumor thickness, ulceration, age, gender, SLN status, CLND status, adjuvant interferon). *p*-values of <0.05 were considered significant.

## 3. Results

We identified 929 melanoma patients [males: 430 (46.3%); females: 499 (53.7%); median age was 58 years, range: 15–90 years)] who had undergone SLNB. Of these 929 patients, 258 (27.8%) had a positive SLN status. Details of demographics and results of univariable statistics regarding the SLN-positive patients (*n* = 258) with or without CLND are listed in Table 1. Median relapse-free survival was 72 months (1–251) and median MSS 96 months (2–251). CLND was performed in 209 of 258 patients (81%) with positive SLN status. In 54 of 209 patients with CLND (25.8%), positive lymph nodes were detected (median = 1.5 (range: 1–28)). Whereas the detected number of positive SLNs was not significantly (*p* = 0.39) associated with MSS, patients with more than one detected positive lymph node on CLND more frequently had unfavorable MSS (*p* = 0.0029). In 49 of 258 SLN-positive patients (19%), CLND was not performed. The reasons for this were diverse, including comorbidities, missing patient consent, and changes in melanoma management. As shown in Table 1, SLN-positive patients without consecutive CLND did not significantly (*p* > 0.05) differ from patients with CLND with respect to important prognostic parameters, including age, gender, tumor thickness, tumor ulceration, capsule infiltration of SLN, invasion level of SLN, disease relapses, and deaths.

**Figure 1:**
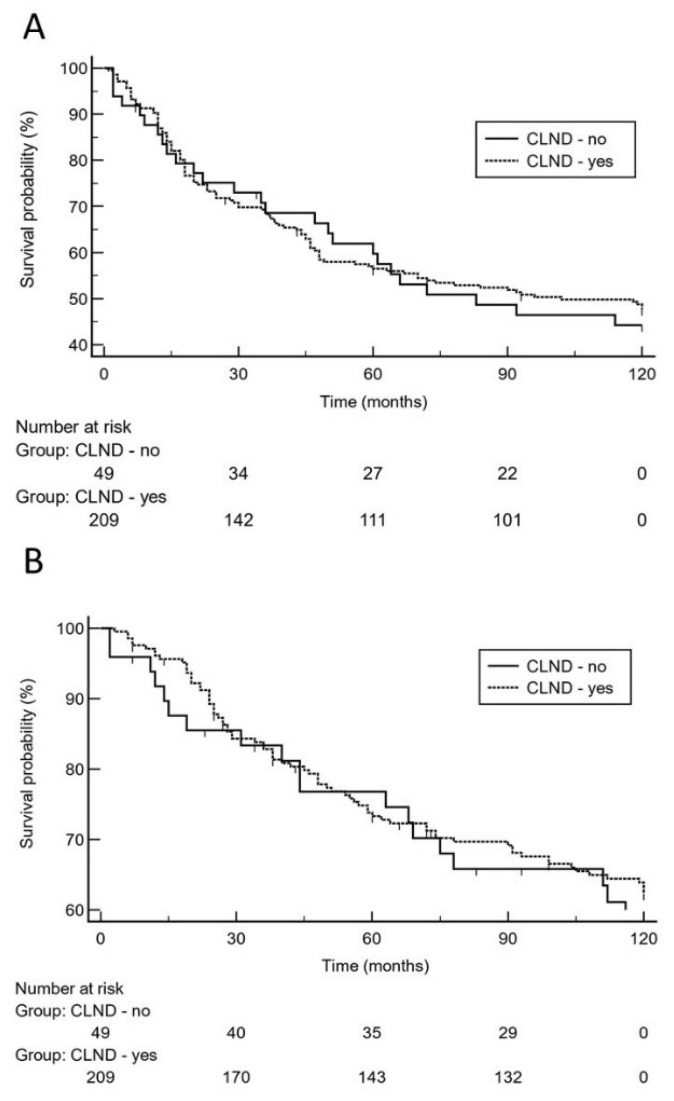
On the basis of 10-year survival data, Kaplan-Meier curves show that there was no significantly increased risk for melanoma relapse (**A**), [hazard ratio: 1.1 (95% CI 0.67 to 1.7)] or decreased melanoma-specific survival (**B**), [hazard ratio: 1.1 (95% CI 0.67 to 1.9)] in patients who did not undergo complete lymph node dissection (CLND) after positive sentinel lymph node biopsy.

The anatomic sites of relapses (local, regional, distant) did not significantly (*p* = 0.52) differ between patients with or without CLND as well. However, there was a significant (*p* = 0.0007) difference between both groups regarding the use of adjuvant interferon therapy, which was more frequently employed in patients with CLND (83.3% vs. 61.2%, Table 1). Overall, there were 25 (51%) relapse events in the 49 patients without CLND vs. 106 (50.7%) relapses in the 209 patients with CLND. Moreover, we observed 19 (38.8%) melanoma-specific deaths in the 49 patients without CLND vs. 75 (35.9%) melanoma-specific deaths in the 209 patients with CLND. Based on the 10-year analysis (Figure 1), there was no significantly increased risk for melanoma relapse in patients who did not undergo CLND after positive SLN biopsy as compared to patients who had undergone CLND [*p* = 0.78; hazard ratio: 1.1 (95% CI 0.67 to 1.7)].

**Figure 2:**
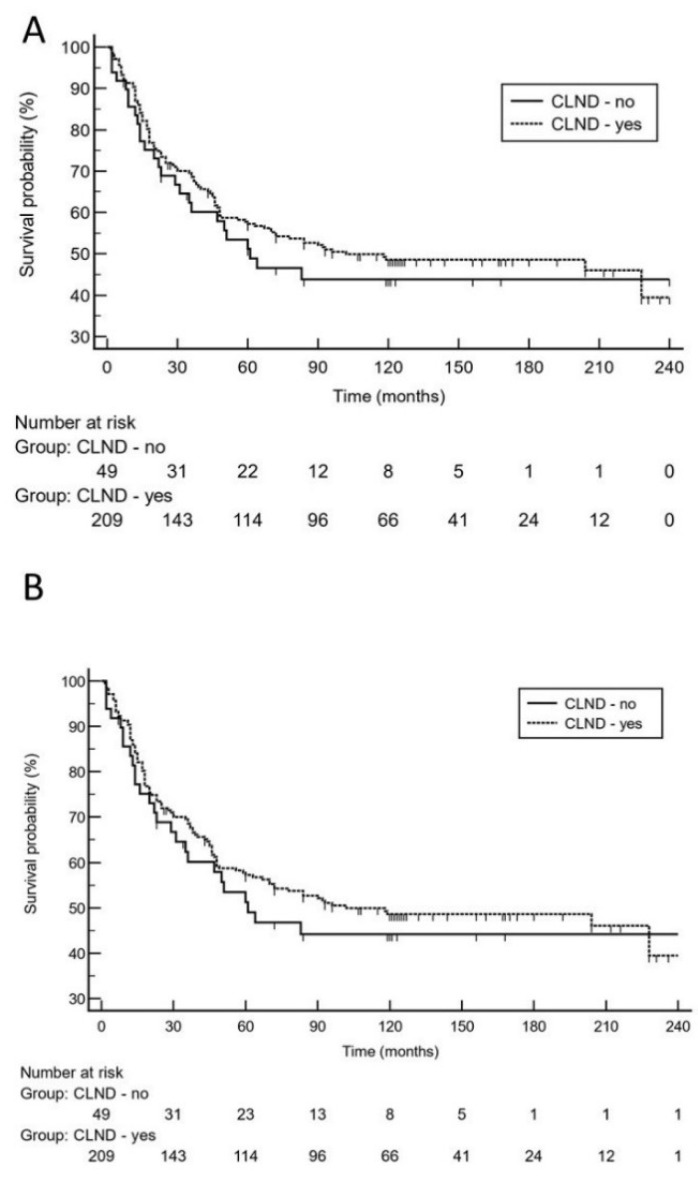
On the basis of 20-year survival data, Kaplan-Meier curves demonstrate that there was no significantly increased risk for late melanoma relapse (**A**), [hazard ratio: 1.2 (95% CI 0.8 to 1.9)] or worse melanoma-specific survival (**B**), [hazard ratio: 1.3 (95% CI 0.8 to 2.3)] in patients who did not undergo complete lymph node dissection (CLND) after positive sentinel lymph node biopsy.

Moreover, 10-year survival analysis did not reveal a significantly increased risk for impaired MSS in patients who did not undergo CLND [*p* = 0.64; hazard ratio: 1.1 (95% CI 0.67 to 1.9)]. On logistic regression analysis, high tumor thickness (*p* = 0.0004; odds ratio: 2.6, 95% CI 1.5 to 4.5) and higher age (*p* = 0.0013; odds ratio: 2.5, 95% CI 1.4 to 4.4) were independent predictors for melanoma relapse. With respect to the risk of dying from melanoma, high tumor thickness (*p* = 0.012; odds ratio: 2.2, 95% CI 1.2 to 3.9) and tumor ulceration (*p* = 0.032; odds ratio: 1.8, 95% CI 1.1 to 3.1) were independent predictors. Notably, positive SLN status did not remain in the regression model. Using the 10-year dataset (Table 2), low tumor thickness, ulceration, and absence of adjuvant interferon therapy remained in the Cox proportional-hazards regression model as significant predictors for melanoma relapse and MSS, respectively. CLND status did not remain in the model (*p* > 0.1). Using the 20-year follow-up data (Table 2), again, low tumor thickness, ulceration, and absence of adjuvant interferon therapy were the only significant predictors for melanoma relapse and MSS, respectively. The status of CLND did not remain in the regression model (*p* > 0.1). On 20-year analysis, Kaplan-Meier curves illustrate that there was no significantly increased risk for late melanoma relapse or Melanoma-specific death in patients who did not undergo CLND after positive sentinel lymph node biopsy (Figure 2).

## 4. Discussion

Melanoma surgery has been changed during the last decades. For example, safety margins in the excision of primary melanomas are currently considerably smaller than in previous times. Furthermore, the clinical value of CLND for melanoma patients with SLN micrometastases has been put into perspective within the last decade. Since about 80% of patients with SLN micrometastases have their nodal disease exclusively confined to the SLN, the majority of SLN-positive patients who are treated with CLND are exposed to potential complications of the CLND procedure when they probably cannot benefit from the additional surgical intervention. By treating with immediate CLND for SLN micrometastases, one may prevent the development of palpable metastases in the draining basin allowing for increased regional tumor control. But only patients with micrometastases beyond the SLN—which only applies to about 20% of SLN-positive cases—benefit from CLND. In contrast, patients with additional non-SLN micrometastases have worse survival rates, and thus having the knowledge about the status of non-SLN tumor load potentially provides prognostic information [14,15,16,17]. Our data confirm the relatively low number of non-SLN micrometastases detected on CLND in 25.8% of cases.

Recently, two pivotal randomized controlled trials have addressed the value of CLND in almost 2500 melanoma patients with SLN micrometastases [5,6,7]. In both of these investigations, patients with a positive SLN were prospectively randomized to performance of CLND versus ultrasound observation, and CLND only in case of later nodal recurrence. For patients with a positive SLN, these studies do not demonstrate a significant survival benefit of performing a CLND versus nodal observation [5,6,7]. As in several previously published retrospective trials [9,10,18,19,20], our patient collective from a single cancer center did not significantly differ with respect to most known prognostic factors (age, tumor thickness, ulceration). Since we did not observe significant differences between the CLND and non-CLND groups with respect to important prognostic parameters, except for adjuvant interferon, we refrained from retrospective matched-pair approaches performed by other researchers. Notably, our investigation also took a detailed analysis of important prognostic parameters in the SLN into account [15,16]. Hence, we have included in our analysis data on capsule infiltration in the SLN, tumor invasion level, and, in particular, number of positive SLN. The latter was considered only in two previous retrospective trials [9,20]. We did not observe significant differences between both groups for these important prognostic parameters. In a survey from Australia and New Zealand published in 2021, replies were received from respondents in 17 countries [17]. Of these, 97% were familiar with the pivotal clinical trials published by Faries et al. [5] and Leiter et al. [6] In the survey, 5% of respondents reported routinely recommending CLND and 55% recommend CLND in selected cases. Downs et al. [17] found that respondents were most likely to recommend CLND when multiple SLNs were positive.

In a retrospective investigation performed by van der Ploeg et al. [9] in 1174 patients with SLN-positive melanoma 1113 patients with CLND were compared to 61 patients without CLND. They observed that CLND had no positive effect on MSS in univariate and multivariate statistics. Similar data were reported in a single center study by Satzger et al. [10] who compared SLN-positive melanoma patients (*n* = 305) with or without immediate CLND. They found that patients with a minimum tumor burden (<0.1 mm) in the SLN did not significantly benefit from CLND. Even in patients with higher tumor load in the SLN, CLND did not turn out to be a prognostic marker in multivariate statistics [10]. In contrast, Lee et al. [20] also retrospectively studied 471 patients with positive SLN biopsy including 375 (79.6%) who underwent CLND and 96 (20.4%) who did not undergo CLND. The groups were comparable except that the CLND group was younger and had more SLN removed. Interestingly, MSS of patients in the CLND group was significantly (*p* = 0.015) superior to the observation group with a 10-year survival rate of 66.8 vs. 48.3%. On multivariate analysis, CLND was also associated with improved MSS (hazard ratio 0.60, 95% CI 0.40–0.89, *p* = 0.011) and lower nodal recurrence (hazard ratio 0.46, 95% CI 0.24–0.86, *p* = 0.016) [20].

Indeed, our 10-year survival data give support to the results of the two randomized trials and most retrospective observations, indicating that SLN-positive patients without immediate CLND do not perform worse with respect to MSS compared to SLN-positive patients with CLND. By contrast, we did also not observe a decreased relapse-free 10-year survival in non-CLND patients as found by other research groups [5,6] In contrast to previous studies, we also performed a 20-year survival analysis. Hence, we could confirm the 10-year survival data of the present and previous studies by long-term follow-up analysis, showing that waiving CLND did not turn out to be a significant predictor for melanoma relapse and worse MSS. Of note, patients with CLND had significantly more frequently adjuvant interferon therapy compared with the patients without CLND. The only significant factor differing between both groups studied. Moreover, our multivariable analysis revealed that absence of adjuvant interferon therapy was a significant independent predictor for worse outcomes. This observation may indicate that there is still a role for adjuvant interferon in melanoma, even though the efficacy of adjuvant interferon in this setting is considered very limited.

From an immunological point of view, one may speculate that waiving CLND is of clinical benefit [21,22,23]. The SLN status is considered as an indicator for the metastatic capacity of the tumor [21,22,23]. Melanomas may suppress immune activity not only within the tumor, but also in the draining of lymphatic tissues as is evidenced by reduced counts and/or clonality of tumor reactive cytotoxic T cells in the SLN. Thus, the immune status of the SLN seems to depend on factors transferred from the primary tumor. If immunosuppression induced by the primary tumor prevents destruction of nodal micrometastases, removal of the primary should allow subsequent reactivation toward a more active immune status that—in the best case—allows immunologic elimination of tumor cells [21,22,23]. Hence, regional lymphatic immune processes may also explain at least in part why SLN-positive melanoma patients do not perform worse without immediate CLND even though 20% of these patients likely have occult non-SLN metastases.

Nevertheless, similar to several of the aforementioned studies, there are also several limitations to the present study, which was retrospective in nature and reflects the referral bias and practice pattern of a single cancer center [20]. A selection bias for those who underwent primary observation compared to those who underwent CLND may have occurred, even though there were no significant differences with respect to important prognostic parameters. Moreover, the sample size included in the present study was relatively small and disbalanced.

## 5. Conclusions

Our 10-year data give support to previous investigations indicating that waiving CLND in patients with SLN micrometastases does not affect MSS. More importantly, our long-term follow-up data over a 20-year period confirm for the first time the 10-year survival data of previous investigations.

## Figures and Tables

**Table 1:** Outcome of melanoma patients (*n* = 258) following sentinel lymph node (SLN) biopsy with (*n* = 209) or without (*n* = 49) immediate complete lymph node dissection (CLND) on the basis of positive SLN status.

Parameters	CLND Not Performed (*n* = 49)	CLND Performed (*n* = 209)	*p*-Value (Mann-Whitney, Chi^2^ test)
Age			=0.45
<65 years	31 (63.3%)	144 (68.9%)	
≥65 years	18 (36.7%)	65 (31.1%)	
Gender			=0.81
F	23 (46.9%)	102 (48.8%)	
M	26 (53.1%)	107 (51.2%)	
Tumor thickness (mm)	2.4 (0.96–11)	2.5 (0.74–24)	=0.55
High-risk melanoma (>2 mm thickness)			=0.46
No	16 (32.7%)	80 (38.3%)	
Yes	33 (67.3%)	129 (61.7%)	
Ulceration			=0.10
No	23 (46.9%)	125 (59.8%)	
Yes	26 (53.1%)	84 (40.2%)	
More than 1 positive SLN			=1.0
No	44 (88.6%)	189 (89.4%)	
Yes	5 (11.4%)	20 (10.6%)	
Capsule infiltration in SLN ^§^			=0.72
No	41 (89.1%)	188 (90.8%)	
Yes	5 (10.9%)	19 (9.2%)	
SLN invasion level (mm)	0.7 (0.1–3.4)	0.54 (0.05–32)	=0.83
Adjuvant interferon			=0.0007 *
No	19 (38.8%)	35 (16.7%)	
Yes	30 (61.2%)	174 (83.3%)	

^§^ unknown data of 5 cases not included, * statistically significant.

**Table 2:** Cox proportional-hazards regression model for 10-year as well as 20-year melanoma relapse (MR) and melanoma-specific survival (MSS) in 258 patients with positive sentinel lymph node (SLN) biopsy with or without subsequent complete lymph node dissection (CLND).

Variables Included	10-Year Survival				20-Year Survival			
	MR		MSS		MR		MSS	
	HR (95% CI)	*p*-Value	HR (95% CI)	*p*-Value	HR (95% CI)	*p*-Value	HR (95% CI)	*p*-Value
Tumor thickness < 2 mm	0.52 (0.34–0.78)	=0.0018	0.54 (0.33–0.9)	=0.017	0.49 (0.32–0.74)	=0.0008	0.52 (0.31–0.86)	=0.012
Ulceration	1.46 (1.01–2.1)	=0.041	1.7 (1.1–2.7)	=0.012	1.5 (1.1–2.1)	=0.033	1.7 (1.2–2.8)	=0.0082
No adjuvant interferon	1.7 (1.1–2.7)	=0.014	1.8 (1.2–2.9)	=0.0098	1.9 (1.2–2.8)	=0.0027	2.1 (1.3–3.3)	=0.0019
CLND status SLN status Age Gender	n.a.	n.a.	n.a.	n.a.	n.a.	n.a.	n.a.	n.a.

n.a. = not applicable as the variables did not remain in the regression model (*p* > 0.1).

## Data Availability

Derived data supporting the findings of this study are available from the corresponding author on reasonable request.

## References

[1] S3-Leitlinie zur Diagnostik, Therapie und Nachsorge des Melanoms Version 3.3—Juli 2020 AWMF-Register-Nummer: 032/024OL. https://www.awmf.org/leitlinien/detail/ll/032-024OL.html.

[2] Morton D.L., Thompson J.F., Cochran A.J., Mozzillo N., Nieweg O.E., Roses D.F., Hoekstra H.J., Karakousis C.P., Puleo C.A., Coventry B.J. (2014). Final trial report of sentinel-node biopsy versus nodal observation in melanoma. N. Engl. J. Med..

[3] Ferrara G., Partenzi A., Filosa A. (2018). Sentinel Node Biopsy in Melanoma: A Short Update. Dermatopathology.

[4] Nieweg O.E., Cooper A., Thompson J.F. (2018). The role of sentinel lymph node biopsy as a staging procedure in patients with melanoma—A critical appraisal. Australas J. Dermatol..

[5] Faries M.B., Thompson J.F., Cochran A.J., Andtbacka R.H., Mozzillo N., Zager J.S., Jahkola T., Bowles T.L., Testori A., Beitsch P.D. (2017). Completion dissection or observation for sentinel-node metastasis in melanoma. N. Engl. J. Med..

[6] Leiter U., Stadler R., Mauch C., Hohenberger W., Brockmeyer N., Berking C., Sunderkötter C., Kaatz M., Schulte K.W., Lehmann P. (2016). Complete lymph node dissection versus no dissection in patients with sentinel lymph node biopsy positive melanoma (DeCOG-SLT): A multicentre, randomised, phase 3 trial. Lancet Oncol..

[7] Leiter U., Stadler R., Mauch C., Hohenberger W., Brockmeyer N.H., Berking C., Sunderkötter C., Kaatz M., Schatton K., Lehmann P. (2019). German Dermatologic Cooperative Oncology Group. Final Analysis of DeCOG-SLT Trial: No Survival Benefit for Complete Lymph Node Dissection in Patients With Melanoma With Positive Sentinel Node. J. Clin. Oncol..

[8] Bilimoria K.Y., Balch C.M., Bentrem D.J., Talamonti M.S., Ko C.Y., Lange J.R., Winchester D.P., Wayne J.D. (2008). Complete lymph node dissection for sentinel node-positive melanoma: Assessment of practice patterns in the United States. Ann. Surg. Oncol..

[9] van der Ploeg A.P., van Akkooi A.C., Rutkowski P., Cook M., Nieweg O.E., Rossi C.R., Testori A., Suciu S., Verhoef C., Eggermont A.M. (2012). European Organization for Research and Treatment of Cancer Melanoma Group. Prognosis in patients with sentinel node-positive melanoma without immediate completion lymph node dissection. Br. J. Surg..

[10] Satzger I., Meier A., Zapf A., Niebuhr M., Kapp A., Gutzmer R. (2014). Is there a therapeutic benefit of complete lymph node dissection in melanoma patients with low tumor burden in the sentinel node?. Melanoma Res..

[11] Gambichler T., Scholl L., Stücker M., Bechara F.G., Hoffmann K., Altmeyer P., Othlinghaus N. (2013). Clinical characteristics and survival data of melanoma patients with nevus cell aggregates within sentinel lymph nodes. Am. J. Clin. Pathol..

[12] Gambichler T., Bünnemann H., Scheel C.H., Bechara F.G., Stücker M., Stockfleth E., Becker J.C. (2020). Does very early timing of lymph node surgery after resection of the primary tumour improve the clinical outcome of patients with melanoma?. Clin. Exp. Dermatol..

[13] Scolyer R.A., Murali R., McCarthy S.W., Thompson J.F. (2008). Pathologic examination of sentinel lymph nodes from melanoma patients. Semin. Diagn. Pathol..

[14] Han D., van Akkooi A.C.J., Straker R.J., Shannon A.B., Karakousis G.C., Wang L., Kim K.B., Reintgen D. (2021). Current management of melanoma patients with nodal metastases. Clin. Exp. Metastasis.

[15] Satzger I., Völker B., Al Ghazal M., Meier A., Kapp A., Gutzmer R. (2007). Prognostic significance of histopathological parameters in sentinel nodes of melanoma patients. Histopathology.

[16] Kretschmer L., Mitteldorf C., Hellriegel S., Leha A., Fichtner A., Ströbel P., Schön M.P., Bremmer F. (2021). The sentinel node invasion level (SNIL) as a prognostic parameter in melanoma. Mod. Pathol..

[17] Downs J.S., Subramaniam S., Henderson M.A., Paton E., Spillane A.J., Mathy J.A., Gyorki D.E. (2021). A survey of surgical management of the sentinel node positive melanoma patient in the post-MSLT2 era. J. Surg. Oncol..

[18] Wong S.L., Morton D.L., Thompson J.F., Gershenwald J.E., Leong S.P., Reintgen D.S., Gutman H., Sabel M.S., Carlson G.W., McMasters K.M. (2006). Melanoma patients with positive sentinel nodes who did not undergo completion lymphadenectomy: A multi-institutional study. Ann. Surg. Oncol..

[19] Kingham T.P., Panageas K.S., Ariyan C.E., Busam K.J., Brady M.S., Coit D.G. (2010). Outcome of patients with a positive sentinel lymph node who do not undergo completion lymphadenectomy. Ann. Surg. Oncol..

[20] Lee D.Y., Lau B.J., Huynh K.T., Flaherty D.C., Lee J.H., Stern S.L., O’Day S.J., Foshag L.J., Faries M.B. (2016). Impact of Completion Lymph Node Dissection on Patients with Positive Sentinel Lymph Node Biopsy in Melanoma. J. Am. Coll. Surg..

[21] Grotz T.E., Mansfield A.S., Jakub J.W., Markovic S.N. (2012). Regional lymphatic immunity in melanoma. Melanoma Res..

[22] Straten Pt Dahl C., Schrama D., Pedersen L.Ø., Andersen M.H., Seremet T., Bröcker E.B., Guldberg P., Becker J.C. (2006). Identification of identical TCRs in primary melanoma lesions and tumor free corresponding sentinel lymph nodes. Cancer Immunol. Immunother..

[23] Cochran A.J., Huang R.R., Lee J., Itakura E., Leong S.P., Essner R. (2006). Tumour-induced immune modulation of sentinel lymph nodes. Nat. Rev. Immunol..

